# Case–Case Analysis Using 7 Years of Travelers' Diarrhea Surveillance Data: Preventive and Travel Medicine Applications in Cusco, Peru

**DOI:** 10.4269/ajtmh.16-0633

**Published:** 2017-05-03

**Authors:** Mary Carol Jennings, Drake H. Tilley, Sarah-Blythe Ballard, Miguel Villanueva, Fernando Maldonado Costa, Martha Lopez, Hannah E. Steinberg, C. Giannina Luna, Rina Meza, Maria E. Silva, Robert H. Gilman, Mark P. Simons, Ryan C. Maves, Miguel M. Cabada

**Affiliations:** 1 Department of International Health, Johns Hopkins Bloomberg School of Public Health, Baltimore, Maryland; 2 Preventive Medicine, Johns Hopkins Bloomberg School of Public Health, Baltimore, Maryland; 3 Bacteriology Department, Naval Medical Research Unit No. 6, Lima, Peru; 4 Parasitology Department, Naval Medical Research Unit No. 6, Lima, Peru; 5 Asociación Benéfica PRISMA, Lima, Peru; 6 Hematology Department, Hospital Universitario de la Princesa, Madrid, Spain; 7 Department of Epidemiology, Royal Tropical Institute, Amsterdam, The Netherlands; 8 Department of Internal Medicine, University of Texas Medical Branch, Galveston, Texas; 9 Collaborative Research Center, Universidad Peruana Cayetano Heredia, Cusco, Peru; 10 Virology Department, Naval Medical Research Unit No. 6, Lima, Peru; 11 Division of Infectious Diseases, Naval Medical Center San Diego, San Diego, California

## Abstract

In Cusco, Peru, and South America in general, there is a dearth of travelers' diarrhea (TD) data concerning the clinical features associated with enteropathogen-specific infections and destination-specific risk behaviors. Understanding these factors would allow travel medicine providers to tailor interventions to patients' risk profiles and travel destination. To characterize TD etiology, evaluate region-specific TD risk factors, and examine relationships between preventive recommendations and risk-taking behaviors among medium- to long-term travelers' from high-income countries, we conducted this case–case analysis using 7 years of prospective surveillance data from adult travelers' presenting with TD to a physician in Cusco. At the time of enrollment, participants provided a stool sample and answered survey questions about demographics, risk behaviors, and the clinical features of illness. Stool samples were tested for norovirus (NV), bacteria, and parasites using conventional methods. Data obtained were then analyzed using case–case methods. NV (14%), enterotoxigenic *Escherichia coli* (11%), and *Campylobacter* (9%), notably ciprofloxacin-resistant *Campylobacter*, were the most frequently identified pathogens among adults with TD. Coinfection with multiple enteropathogens occurred in 5% of cases. NV caused severe disease relative to other TD-associated pathogens identified, confining over 90% of infected individuals to bed. Destination-specific risk factors include consumption of the local beverage “chicha,” which was associated with *Cryptosporidium* infection. Preventive interventions, such as vaccines, directed against these pathogens could significantly reduce the burden of TD.

## Introduction

Cusco, Peru, is a popular travel destination in the Andes mountain range that is famous for its archaeological remains, including Machu Picchu. Previous studies have identified Cusco as a destination associated with a high risk of acute travelers' diarrhea (TD), with U.S. travelers at higher risk than those from other countries (adjusted odds ratio [OR] = 1.28; 95% confidence interval [CI] = 1.09–1.50).^1^

However, in Cusco, and South America in general, the specific etiologies of TD have not been well evaluated, and the clinical features associated with pathogen-specific infections, along with destination-specific risk behaviors, remain largely uncharacterized. Understanding these factors would allow travel medicine providers to tailor interventions to patients' risk profiles and travel destination.^2^

Anticipatory guidance and interventions (e.g., immunizations and prophylaxis) aim to decrease the incidence of travel-related injury and illness. Previous work shows that 94% of travelers to Cusco seek some sort of pretravel preventive guidance.^3^

However, North American travelers to Peru are less likely than western European travelers to seek pretravel preventive medical advice from a travel medicine practitioner (37% versus 45.8%, *P* < 0.01) and from a health-care professional (52.0% versus 67.1%, *P* < 0.01).^3^,^4^

Further, current health-related guidelines and dietary precautions have little evidence of effect on the incidence of TD.^5^–^7^

Although most patients with TD experience spontaneous symptom resolution, some may experience symptoms for weeks,^8^ and up to half of travelers change travel plans as a result of symptoms.^5^

Of note, immunocompromised patients from high-income countries are traveling at increasing rates, and their increased risk for developing disease highlights the importance of providing effective preventive recommendations and measures for such complex patients.^9^ The development of subsequent irritable bowel syndrome has become an increasingly recognized risk in the past decade,^10^ reported by up to 1/5 of returning travelers with TD.^5^

Thus, the benefits of reducing TD incidence extend beyond the travel period.

Traditionally, TD has been difficult to characterize because most data are derived from sentinel surveillance systems, which often lack denominator data and asymptomatic controls for comparison. A case–case approach allows a comparison of symptom severity between pathogens identified in the stool, although it lacks mechanisms to question if a particular pathogen present in the stool is causally related to diarrheal disease. A case–case approach allows restricted and refined analysis of some unique exposures associated with different pathogens detected through a surveillance system.^11^–^13^

Although the approach reduces selection and recall bias relative to case–control formats^14^ by ensuring that both case and “control” subjects were all affected by similar disease, the design does remain vulnerable to other forms of selection bias, that is, greater selection of infected travelers with severe symptoms compared with milder symptoms. To describe the etiology of medically attended TD, evaluate regional risk factors, and examine relationships between preventive recommendations and risk-taking behaviors among medium- to long-term travelers from high-income countries, we conducted this case–case analysis of 7 years of prospective surveillance data from adults presenting to a physician in Cusco with TD.

## Materials and Methods

### Ethics statement.

This study was approved by the institutional review boards of Naval Medical Research Unit No. 6 and Universidad Peruana Cayetano Heredia. Participants provided written informed consent at the time of enrollment.

### Study population.

Between June 2003 and July 2010, we prospectively enrolled Spanish language students presenting consecutively with acute diarrhea to the Amauta Spanish School's physician. Male and female students, older than 18 years, from countries with low-to-moderate risk of TD, and seeking free medical attention were included.^15^

We defined diarrhea as three or more loose or watery stools, or two loose or watery stools accompanied by nausea, vomiting, abdominal cramping/pain, or tenesmus, within a 24-hour period.^16^,^17^

To reduce confounding by chronic or recently treated diarrhea, students who could not provide a stool sample at the time of presentation, had taken antimicrobial medications within the week prior to presentation, reported chronic diarrhea, or did not speak English were excluded.

### Survey administration, sample collection, pathogen identification, and antimicrobial resistance testing.

Using a standardized form, a trained health-care worker surveyed enrollees to determine age, gender, country of birth, country of residence, lodging, and length of stay in Cusco, and Peru, in addition to TD risk factors, such as history of previous episodes, comorbidities, preventive measures, location and type of foods ingested, and clinical features of the presenting episode. Nonrecommended foods were defined using standard lists of unsafe foods.^2^

We collected survey responses from individual cases while waiting for laboratory results to further reduce information and recall bias. The collection of individual-level data permitted us to control for potential confounding.

Participants provided fresh stool samples for analysis. Upon collection, specimens were kept fresh in preservative-free containers and transported in Cary Blair media to a central reference laboratory (ServiSalud) in Cusco city for bacteria and parasite analyses. On arrival, stool specimens went through a microscopic examination looking for ova and parasites using saline wet preparations to detect protozoa and helminth infections. The Ritchie method^18^ was used to identify *Giardia* spp. Cary Blair specimens were cultured for *Campylobacter*, *Escherichia coli*, *Salmonella*, *Shigella*, *Aeromonas*, and *Plesiomonas* using conventional microbiologic techniques.^18^–^20^

We performed polymerase chain reaction (PCR) on five lactose-fermenting colonies morphologically resembling *E. coli* to identify heat labile and stable enterotoxigenic (ETEC), enteropathogenic (EPEC), and enteroaggregative (EAEC) *E. coli*.^21^

Antibiotic susceptibility testing was performed using the disk diffusion method according to the Clinical and Laboratory Standards Institute (CLSI) guidelines.^22^

The isolates were tested against the most commonly used antimicrobial agents: ampicillin, amoxicillin–clavulanic acid, cefazolin, ceftriaxone, erythromycin, azithromycin, nalidixic acid, ciprofloxacin, gentamicin, sulfamethoxazole–trimethoprim, and tetracycline.^22^

In the case of azithromycin, because of the absence of an established breakpoint, minimal inhibitory concentration was also determined by the agar dilution method according to CLSI guidelines^22^ on all isolates with an inhibitory halo < 15 mm.^23^

We combined intermediate and resistant isolates for analysis purposes. To determine Enterobacteriaceae susceptibility given lack of breakpoints published in CSLI, our laboratory used *Staphylococcus aureus* for quality control, and then established a resistance cutoff of 16 mm^24^ with a susceptible strain of *E. coli* 25922.^22^,^25^–^28^

After preparing fresh specimens for parasite identification, the remaining stool was frozen and sent to Naval Medical Research Unit No. 6 (NAMRU-6) in Lima, Peru, for additional parasite testing using microscopy and commercial enzyme-linked immunosorbent assay (ELISA) kits for *Entamoeba histolytica* (TechLabs, Blacksburg, VA), *Giardia lamblia* (BioTech Trading Partners), and *Cryptosporidium* (BioTech Trading Partners, Encinitas, CA). *Cryptosporidium*-positive results were confirmed by PCR. Samples positive by microscopy but negative by ELISA were considered positive; in any further cases of discrepancy between results of pathogen identification at ServiSalud and NAMRU-6, NAMRU-6 results were considered confirmatory. A suspension of each stored stool specimen (10% w/v) was prepared in phosphate-buffered saline and RNA extracted using the Qiagen QIAmp Viral RNA Kit (Valencia, CA) in accordance with the manufacturer's instructions. Viral RNA was tested for Norovirus (NV) GI and GII by real-time PCR.^29^

A sample was considered positive if the negative control did not exhibit fluorescent curves. The threshold cycle for the sample was at least 37 for NV GI and 39 for GII.

### Statistical analysis.

Statistical analysis was performed using Stata version 13.0 (StataCorp LP, College Station, TX). By convention, *P* values < 0.05 were considered statistically significant. Exploratory analysis of participant demographics, diarrhea risk factors, and clinical features of illness was performed using the two-tailed Student's *t* test to compare continuous variable means for normally distributed variables, Wilcoxon rank-sum tests for nonparametric continuous variables, and Fisher's exact test to compare proportions. We used dichotomous variables to characterize any versus no exposure to individual diarrhea risk factors. After excluding individuals coinfected with more than one pathogen, we performed a case–case analysis to examine the association of single-pathogen infections with dietary risk factors, according to the methods of Wilson and others.^30^

It is standard practice in a case–case analyses to take the most prevalent or most frequently notified cause as the referent or comparison group.^30^

We selected ETEC as the case–case analysis reference group because it is the most commonly implicated pathogen worldwide,^2^ has the added benefit of being the most frequently identified cause of TD in Latin America, and has well characterized risk factors and clinical feature profiles.^17^

We considered age and gender to be confounders a priori,^13^,^14^ and controlled for them using multiple logistic regression. Exploratory analysis did not identify additional potential confounding factors.

## Results

### Demographics.

During the 7-year passive surveillance period, 230 adults (66% female) aged 18–76 (median = 24, interquartile range [IQR] = 7) years with diarrhea were enrolled in the study (Table 1). Participants were permanent residents of 17 countries, 75% were European, 21.3% North American, and 3.5% Australian. The mean length of stay in Cusco was 45 days (median = 28, range = 1–548, IQR = 52). The diarrhea incidence among cases was 2.2 episodes per 90 person-days (230/9,417.5) in Cusco.

### Reported diarrhea risk factors.

During the week before presenting with diarrhea, 47% of participants ate the majority of meals in a restaurant, 15.2% in a friend's home, 13% in a hotel, and 24.8% in an “other” location (Table 1). All participants reported consuming at least one of the following risky food items time during the week before illness: fruit juice (86%), cheese (78%), raw green vegetables (72%), cold sauces (52%), milk (46%), ice (40%), reheated buffet foods (38%), ice cream (33%), unpeeled fruit (29%), street vendor food (28%), the local drink chicha (25%), and tap water (14%) (Table 2).

Approximately two-thirds (176/230) of study participants reported receiving advice to prevent diarrhea. These individuals reported following this guidance all (46%), some (51%), or none (3%) of the time. Among those who reported always following recommendations, 93% (84/90) reported consuming three or more of the abovementioned foods linked to TD. Among these three stratified risk groups, the majority consumed fruit juice (84%, 86%, and 83%), cheese (73%, 83%, and 83%), or raw green vegetables (58%, 81%, and 83%), and the majority (84%) of the group that reported always following recommendations also consumed at least three foods linked to TD. In addition, 55/230 (24%) study participants took medications to prevent diarrhea, at some time prior to 1 week before enrollment. Of these individuals, 50 provided information on the type of medication used: 32% had taken antibiotics, 4% took bismuth preparations, and 64% took “other” medications.

### Pathogen identification.

A pathogen was identified in 45% of 230 cases (Table 3). NV was identified in 14% of samples (6 GI, 26 GII), ETEC in 11%, *Campylobacter* in 9%, *Shigella* in 6%, EAEC in 4%, *Giardia* in 4%, and *Cryptosporidium* in 3%. No EPEC, *E. histolytica*, *Salmonella*, *Aeromonas*, or *Plesiomonas* were detected. Coinfections with more than one pathogen were identified in 12 (5%) participants. Coinfections were most frequent among cases infected with the parasites *Giardia* (44%, 4/9) and *Cryptosporidium* (43%, 3/7). Coinfections were also found in individuals infected with ETEC (28%, 7/25 ETEC cases) in combination with NV (19%, 6/32 NV cases), EAEC (11%, 1/9 EAEC cases), and *Campylobacter* (10%, 2/20 *Campylobacter* cases). NV and ETEC were most frequently identified together (*N* = 6 coinfected cases), followed by *Cryptosporidium* and *Giardia* (*N* = 2 coinfected cases). Coinfections with the following pathogens were each observed once: EAEC and NV, ETEC and *Giardia*, *Campylobacter* and *Cryptosporidium*, and *Campylobacter* and *Giardia*. *Giardia* was a coinfecting agent in the greatest number of pathogen combinations (three other pathogens). No *Shigella* cases were coinfected. Table 4 shows bacterial antimicrobial resistance patterns, and Table 5 shows the relationship between pathogen prevalence and seasonality.

### TD clinical features and severity.

Gastroenteritis episodes were most frequently associated with watery diarrhea (84%), abdominal cramping/pain (76%), loss of appetite (69%), fatigue (68%), and gas/bloating (63%) (Table 2). Subjective fever was reported in 42% of TD cases, most frequently with *Campylobacter* (75%), *Shigella* (71%), and EAEC (67%). Thirty-four percent of cases reported vomiting. Fifty-six percent of those with EAEC and 50% of those with NV (13/26 GII, 3/6 GI) vomited. Ten percent reported bloody diarrhea, most often with *Shigella* (36%), *Giardia* (22%), *Campylobacter* (20%), ETEC (16%), and NV GII (9%); no NV GI case had bloody diarrhea. These statistics include two individuals who were coinfected with NV GII and ETEC, along with one individual coinfected with *Campylobacter* and *Giardia*. Of the 230 cases, two were hospitalized, 62% stayed in bed, 52% saw a physician, 23% missed a scheduled tour, 13% made a change in travel itinerary, and 18% reported no effect on daily activities. One hospitalized case had no pathogen identified; one was positive for *Cryptosporidium*. Both were females aged 23 who presented in the winter and reported consuming raw green vegetables, fruit juice, and cheese. Neither had chronic gastritis. The case with no pathogen was from Holland, stayed in Cusco for 2 weeks in the winter, and reported she always followed preventive recommendations. The *Cryptosporidium* case was from Belgium, stayed in Cusco for 2 months in the winter, gave no answer regarding following recommendations, and reported consuming chicha. Ninety-one percent of norovirus-associated diarrhea cases were confined to bed. ETEC and *Shigella* cases had the largest proportion of doctors' visits, 60% and 79%, respectively.

### Case–case analysis.

In the unadjusted case–case analysis of diarrhea risk factors, individuals with and without ETEC ate at similar locations and had similar food exposures during the week before illness. However, individuals infected with NV GII were significantly less likely to have consumed reheated buffet food (OR = 0.10, 95% CI = 0.01–0.91), and individuals infected with NV overall were significantly less likely to have consumed food from street vendors (OR = 0.11, 95% CI = 0.01–0.88) relative to those with ETEC, although the buffet food relationship did not persist when the NV strains were combined. Chicha consumption was significantly associated with *Cryptosporidium* (OR = 5.44, 95% CI = 1.1–27.3) and EAEC (OR = 7.33, 95% CI = 1.17–46.1) infections relative to infection with ETEC. Individuals with no pathogen detected were significantly less likely to have consumed ice cream in the past week relative to ETEC (OR = 0.40, 95% CI = 0.17–0.94). Being a permanent resident of the United States or staying in Cusco longer than 1 week were not significantly associated with any pathogen relative to ETEC (data not shown). After adjusting for the potential confounders of age and gender, the statistical significance, OR magnitude, and CI size of these associations remained unchanged (Table 6).

## Discussion

This study is one of the first to evaluate TD among medium- to long-term adult travelers to South America. The 28-day median length of stay reported by participants exceeds the 5-day median stay in Cusco reported previously by travelers^1^ and also the 17-day median stay in a study of global travelers with TD.^31^

Thus, these results may be generalizable to backpackers, international students, individuals involved in volunteer/service programs, and military populations deployed to this region.

We most frequently identified NV (14%), ETEC (11%), and *Campylobacter* (9%) in participants with TD, which differs from the results of a recent review identifying ETEC (33%), NV (15%), and EAEC (13%) as the top three TD pathogens in Latin America.^17^

Although the lack of reported CIs in this comprehensive review does not allow us to comment on the level of significance of this observation, this suggestion of a lower prevalence of ETEC could be due to the inclusion of more severe diarrhea cases among longer-term travelers, along with the absence of NV detection methods in previous TD studies. While cholera vaccination provides some protection against ETEC,^32^ we did not systematically assess this exposure. Of two participants who volunteered vaccination status, ETEC was missing for one and negative for the other, which would not appreciably impact ETEC prevalence. The study methodology was limited by a lack of capability to perform molecular testing for the full range of viral pathogens. For example, we might expect to detect Sapovirus among adult travelers to Cusco,^33^ and data on Rotavirus and Astrovirus could also provide new information if included in future analyses. A recent systematic review of TD from 1973 to 2009 only included one study evaluating NV among travelers to Latin America, with a prevalence of 17% among students traveling to Mexico.^17^

Globally, NV is the most common cause of all varieties of diarrhea, accounting for double the number of cases as ETEC,^34^ and diarrheagenic *E. coli* and NV are commonly identified among Latin American travelers with TD.^35^

Coinfection in this study was common, with half of coinfected TD cases positive for both NV and ETEC, and almost half of coinfected cases testing positive for *Giardia*. NV and ETEC were implicated together in a 2008 gastroenteritis outbreak among U.S. Navy personnel visiting Lima,^36^ and both generally indicate a high level of fecal contamination in the environment. In coinfected travelers, NV is believed to contribute to disease, although detection may be attributable to prolonged viral shedding following a previous symptomatic or asymptomatic NV infection.^17^,^37^,^38^

*Giardia* was also frequently detected in coinfected cases in our study, making it worthwhile to note that a recent debate in the literature emphasizes the commensal gut bacteria role that *Giardia* may play for citizens of countries where it is endemic.^39^–^41^

Excluding participants with chronic diarrhea could have underestimated the contribution of parasites to diarrhea, while excluding those with recent antibiotic use could have impacted the prevalence of antimicrobial resistant pathogens. The exclusion of individuals who could not provide a stool sample at the time of presentation may have biased this study toward the evaluation of cases with particularly severe disease, which assists in the identification of pathogens against which preventive measures might be most impactful. Our population experienced a high rate (62%) of bed confinement, similar to rates reported in military members with TD (52%).^31^

Compared with 4% bed confinement rates among adult Peruvians with NV-associated diarrhea,^42^ NV-infected travelers with TD reported 91% bed confinement, suggesting that preventive measures, such as vaccines, directed against this pathogen could significantly reduce the burden of disease. Given TD's mission aborting potential for military troops,^43^ in whom incapacitation could pose a further threat to life,^33^,^42^ recommendations to military members operating in this geographic area should take NV-associated risks into account.

This study has several travel medicine implications. First, the prevalence of ciprofloxacin-resistant *Campylobacter* in our study reinforces the existing recommendations to empirically treat severe TD with azithromycin.^44^

Our data also suggest that individuals who reported always following preventive recommendations took relatively fewer risks; however, these individuals still engaged in risky behaviors, indicating a possible gap in traveler receipt or understanding of preventive recommendations, or reflecting the difficulty of full compliance with all travel recommendations. In addition to supporting the use of pretravel interventions, such as vaccines, this may indicate that pretravel guidance is not sufficiently region or country specific, as noted by other authors.^31^

Case–case analysis identified ice cream consumption as a risk factor for ETEC compared with cases with no pathogen identified, which, depending on method of milk preparation, could be consistent with the literature showing that ETEC is associated with unpasteurized dairy products.^45^

For instance, the “queso fresco” and other cheeses typical of the Andean diet are often unpasteurized. Case–case analysis identified buffet food and street vendor food as risk factors for ETEC compared with NV GII, as well as an association between chicha with both *Cryptosporidium* and EAEC, which, despite wide CIs, should be considered for future travel medicine recommendations.

Given the case–case methodology, study limitations include the binomial outcome requirement for statistical analysis and the conservative estimates of association it provides given that most TD pathogens share risk factors.^30^

In conclusion, NV was the most frequently identified pathogen among medium- to long-term adult travelers from high-income countries presenting to a physician in Cusco with TD. ETEC and *Campylobacter*, notably ciprofloxacin-resistant *Campylobacter*, were also frequently identified, and coinfection with multiple enteropathogens was common. NV caused severe disease relative to other TD-associated pathogens identified, confining over 90% of infected individuals to bed. Destination-specific risk factors include consumption of the local beverage chicha, which was associated with *Cryptosporidium* infection.

## Figures and Tables

**Table 1:** Demographic characteristics, protective behaviors, and risk factor frequencies for participants

Variable	Mean or frequency	Percent*	Observations	Missing (%)	Standard deviation
Demographic factors					
Age, years	26.9		230	0	9.6
Gender (female)	152	66.1	230	0	
Length of stay in Cusco, days	44.85		210	8.7	60.7
Season (summer/rainy)	100	43.5	230	0	
Country of Residence			230	0	
Netherlands	53	23.0			
United Kingdom	44	19.1			
United States	40	17.4			
Europe, other†	36	15.7			
Switzerland	22	9.6			
Germany	20	8.7			
Canada	9	3.9			
Australia	6	2.6			
Protective factors against TD					
Received advice	176	76.5	230	0	
Followed all recommendations			195	15.2	
Never	6	3.1			
Sometimes	99	50.8			
Always	90	46.2			
Took medications to prevent TD (yes)	55	23.9	230	0	
If yes, what medications?			50	2.2	
Antibiotics	16	32.0			
Other	32	64.0			
Bismuth	2	4.0			
Risk factors					
Location of majority of meals in past week			230	0	
Restaurant	108	47.0			
Other	57	24.8			
Friend's home	35	15.2			
Hotel	30	13.0			

TD = travelers' diarrhea.

* Percent of number of observations for each variable.

† Austria, Belgium, Czech Republic, Denmark, Finland, France, Italy, Israel, Turkey, Spain, and Sweden.

**Table 2:** Signs, symptoms, stool characteristics, and effect of daily activities among those positive for given pathogens, or negative for all pathogens

	Total		Pathogen															
			No Pathogen		EAEC		ETEC		*Campylobacter*		*Cryptosporidium*		*Giardia*		Norovirus		*Shigella*	
	*n*	%	*n*	%	*n*	%	*n*	%	*n*	%	*n*	%	*n*	%	*n*	%	*n*	%
Signs and symptoms																		
Fatigue	156	68	79	63	8	89	18	72	15	75	5	71	5	56	24	75	11	79
Gas or bloating	145	63	79	63	2	22	16	64	11	55	6	86	8	89	18	56	11	79
Nausea	113	49	58	46	6	67	11	44	11	55	5	71	2	22	15	47	9	64
Vomiting	79	34	38	30	5	56	9	36	6	30	3	43	3	33	16	50	3	21
Fever	96	42	45	36	6	67	8	32	15	75	1	14	3	33	13	41	10	71
Abdominal cramping/pain	174	76	88	70	9	100	21	84	14	70	7	100	7	78	27	84	12	86
Loss of appetite	158	69	79	63	7	78	15	60	16	80	6	86	8	89	24	75	12	86
Loss of weight	80	35	44	35	2	22	10	40	7	35	5	71	6	67	11	34	2	14
Characteristics of stool																		
Loose	114	50	72	58	2	22	10	40	7	35	5	71	4	44	12	38	7	50
Bloody	23	10	8	6	1	11	4	16	4	20	0	0	2	22	3	9	4	29
Watery	193	84	99	79	8	89	23	92	19	95	7	100	7	78	31	97	10	71
Mucus	41	18	21	17	1	11	5	20	4	20	1	14	0	0	5	16	5	36
Dietary risks (one or more exposures in the past week)																		
Raw greens/vegetables/salad	160	72	89	74	5	56	18	75	15	75	5	71	6	67	21	68	10	77
Fruit juice	193	86	104	87	8	89	24	96	15	75	6	86	7	78	29	91	11	85
Cold sauce	114	52	62	52	3	33	13	52	9	47	3	43	4	44	18	58	7	54
Cheese	175	78	93	77	7	78	21	84	14	70	6	86	6	67	24	75	13	100
Tap water	30	14	17	15	0	0	2	8	4	20	1	14	0	0	5	16	1	8
Milk	104	46	63	52	2	22	9	36	9	45	1	14	4	44	14	45	6	46
Ice with drinks	89	40	52	43	4	44	10	40	7	37	4	57	1	11	11	34	4	31
Ice cream	74	33	35	29	2	22	12	48	8	44	3	43	1	11	10	31	5	39
Reheated buffet food	83	38	49	43	4	44	6	24	8	42	5	71	5	56	5	17	6	46
Chicha	54	25	30	26	4	50	3	12	4	21	4	57	0	0	5	16	3	23
Street vendor food	63	28	44	37	1	11	7	28	5	25	1	14	0	0	3	9	3	21
Unpeeled fruit	64	29	37	31	3	18	3	12	4	21	1	14	2	22	9	29	4	31
Effect on daily activities																		
No effect	41	18	23	18	1	11	3	12	2	10	2	29	1	11	4	13	0	0
Stay in bed	143	62	66	53	5	56	17	68	17	85	4	57	5	56	29	91	10	71
Change itinerary	30	13	16	13	2	22	2	8	3	15	1	14	1	11	2	6	4	29
Miss tour	52	23	27	22	3	33	3	12	7	35	1	14	1	11	5	16	5	36
See physician	120	52	60	48	5	56	15	60	10	50	2	29	5	56	18	56	11	79
Hospitalized	2	1	1	1	0	0	0	0	0	0	1	14	0	0	0	0	0	0

EAEC = enteroaggregative *Escherichia coli*; ETEC = enterotoxigenic *E. coli*.

**Table 3:** Pathogen counts and frequencies for 230 total samples

	Positive	Percentage of 230	Negative	No. of samples	Total reporting	Tested* (%)
Norovirus†	32	13.9	141	63	167	72.6
Norovirus GI	6					
Norovirus GII	26					
ETEC†	25	10.9	157	48	182	79.1
St+	16					
Lt+	5					
Lt+/St+	4					
*Campylobacter*†	20	8.7	201	9	221	96.1
*jejuni*	16					
*coli*	3					
Other species	1					
*Shigella*†	14	6.1	215	1	229	99.6
*flexneri*	4					
*sonnei*	9					
Other species	1					
EAEC	9	3.9	45	176	54	23.5
*Giardia*	9	3.9	218	3	227	98.7
*Cryptosporidium*	7	3.0	217	6	224	97.4
EPEC	0	0.0	182	48	182	79.1
*Entamoeba histolytica*	0	0.0	227	3	227	98.7
*Salmonella*	0	0.0	229	1	229	99.6
Total cases				0	230	100.0
Any pathogen	104	45.2				
No pathogen	125	54.3				

EAEC = enteroaggregative *Escherichia coli*; EPEC = enteropathogenic *E. coli*.

* Percentage of samples tested for specific pathogen.

† Subcategories shown are included in the total pathogen counts given.

**Table 4:** Antibiogram by isolate and percent susceptibility

	*n*	AM	AMC	CF	CRO	E	AZM	NA	CIP	GM	SXT	TE
*Campylobacter coli*	3	33	100	0	33	33	100	33	33	100	0	100
*Campylobacter jejuni*	16	56	100	38	13	88	100	25	25	100	0	44
Enteroaggregative *Escherichia coli*	9	56	89	67	89	89	67	100	100	100	78	78
Enterotoxigenic E. coli*	27	59	89	63	100	4	78	67	93	96	59	59
*Shigella flexneri* (Group B)	4	50	75	25	100	0	100	100	100	100	25	0
*Shigella sonnei* (Group D)	9	0	22	67	100	0	56	89	100	100	0	0

AM = ampicillin; AMC = amoxicillin/clavulanic acid; AZM = azithromycin; CF = cefalotin; CIP = ciprofloxacin; CRO = ceftriaxone; E = erythromycin; GM = gentamycin; NA = nalidixic acid; SXT = sulfamethoxazole/trimethoprim; TE = tetracycline.

* Two different resistance patterns were detected in two of the 27 samples and included in the table (*N* = 27).

**Table 5:** Season of presentation by pathogen cases*

	Summer/rainy			Winter/dry			*P* value†
	Pathogen-specific cases	Total cases	Pathogen as % total cases	Pathogen-specific cases	Total cases	Pathogen as % total cases	
EAEC	**6**	82	7.3	**3**	100	3.0	0.072
ETEC	**18**	82	22.0	**7**	100	7.0	0.005
*Campylobacter*	**5**	95	5.3	**15**	126	11.9	0.102
*Cryptosporidium*	**2**	98	2.0	**5**	126	4.0	0.472
*Giardia*	**5**	100	5.0	**4**	127	3.1	0.512
Norovirus GI	**3**	72	4.2	**3**	95	3.2	1.000
Norovirus GII	**17**	72	23.6	**9**	95	9.5	0.017
Norovirus	**20**	72	27.8	**12**	95	12.6	0.017
*Shigella*	**6**	100	6.0	**8**	129	6.2	1.000
No pathogen	**46**	100	46.0	**79**	129	61.2	0.024
Overall	100			130			

EAEC = enteroaggregative *Escherichia coli*; ETEC = enterotoxigenic *E. coli*.

* Summer/rainy season refers to participation in study during November through April, and winter/dry season during May through October.

† Fisher's exact, two-sided χ^2^ of the difference in proportion of season for each pathogen outcome. *P* values < 0.05 are shown in bold.

**Table 6:** Reported dietary risk factor exposure and ORs for different enteric diseases*

	*n*‡	Reported exposure to risk factor				Crude OR			Adjusted OR†		
		Yes	No	Reporting risk (%)	Unknown§ (%)	OR	95% CI	*P* value	OR	95% CI	*P* value
Majority of past week's meals consumed in friend's home, restaurant, or “other,” compared with hotel											
*Campylobacter*	64	55	9	85.9	0.0	0.30	0.07–1.27	0.10	0.31	0.07–1.43	0.13
*Cryptosporidium*	76	66	10	86.8	0.0	***			***		
EAEC	34	30	4	88.2	0.0	1.09	0.1–12.1	0.94	1.04	0.07–15.1	0.98
*Giardia*	78	67	11	85.9	0.0	1.17	0.13–10.5	0.89	1.65	0.14–19.4	0.69
Norovirus GI	70	59	11	84.3	0.0	0.93	0.1–8.79	0.95	0.78	0.08–7.66	0.83
Norovirus GII	76	66	10	86.8	0.0	***			***		
Norovirus	82	71	11	86.6	0.0	5.43	0.66–44.9	0.12	5.42	0.59–49.4	0.13
*Shigella*	72	62	10	86.1	0.0	***			***		
No pathogen detected	151	129	22	85.4	0.0	0.73	0.2–2.67	0.63	0.76	0.21–2.83	0.69
ETEC	182	159	23	87.4	0.0	1.0 (ref)			1.0 (ref)		
Consumed raw green vegetables or salads in past week											
*Campylobacter*	61	46	15	75.4	4.7	0.97	0.28–3.34	0.96	0.12	0.32–4.69	0.77
*Cryptosporidium*	73	54	19	74.0	3.9	0.87	0.15–4.89	0.87	0.67	0.11–3.91	0.65
EAEC	33	23	10	69.7	2.9	0.42	0.08–2.08	0.29	0.44	0.08–2.43	0.34
*Giardia*	75	55	20	73.3	3.8	0.57	0.12–2.63	0.47	0.29	0.05–1.71	0.17
Norovirus GI	68	52	16	76.5	2.9	***			***		
Norovirus GII	73	52	21	71.2	3.9	0.44	0.15–1.32	0.14	0.33	0.09–1.18	0.09
Norovirus	79	58	21	73.4	3.7	0.67	0.24–1.92	0.46	0.56	0.17–1.78	0.32
*Shigella*	69	52	17	75.4	4.2	1.11	0.27–4.62	0.89	1.10	0.25–4.9	0.90
No pathogen detected	146	107	39	73.3	3.3	1.08	0.41–2.83	0.87	1.07	0.41–2.82	0.89
ETEC	177	126	51	71.2	2.7	1.0 (ref)			1.0 (ref)		
Consumed fruit juice in past week											
*Campylobacter*	62	53	9	85.5	3.1	0.32	0.08–1.34	0.12	0.26	0.06–1.21	0.09
*Cryptosporidium*	74	63	11	85.1	2.6	1.05	0.11–9.7	0.96	1.12	0.11–11.8	0.92
EAEC	34	32	2	94.1	0.0	0.33	0.02–5.97	0.46	0.28	0.01–8.64	0.47
*Giardia*	76	65	11	85.5	2.6	0.46	0.08–2.63	0.38	0.66	0.1–4.55	0.67
Norovirus GI	69	58	11	84.1	1.4	0.33	0.05–2.1	0.24	0.23	0.03–1.81	0.16
Norovirus GII	75	65	10	86.7	1.3	3.72	0.44–31.4	0.23	9.11	0.86–95.8	0.07
Norovirus	81	69	12	85.2	1.2	1.50	0.37–6.08	0.57	2.55	0.53–12.2	0.24
*Shigella*	70	60	10	85.7	2.8	0.90	0.17–4.83	0.90	0.87	0.15–4.94	0.87
No pathogen detected	146	128	18	87.7	3.3	0.54	0.12–2.52	0.43	0.55	0.12–2.56	0.44
ETEC	178	157	21	88.2	2.2	1.0 (ref)			1.0 (ref)		
Consumed cold sauce in the past week											
*Campylobacter*	60	31	29	51.7	6.3	0.78	0.26–2.31	0.65	0.85	0.28–2.59	0.78
*Cryptosporidium*	72	38	34	52.8	5.3	0.64	0.13–3.1	0.58	0.59	0.12–2.95	0.52
EAEC	34	16	18	47.1	0.0	0.46	0.09–2.27	0.34	0.38	0.07–2.18	0.28
*Giardia*	74	39	35	52.7	5.1	0.50	0.11–2.27	0.37	0.35	0.07–1.86	0.22
Norovirus GI	67	32	35	47.8	4.3	0.52	0.09–3.03	0.47	0.54	0.09–3.28	0.50
Norovirus GII	72	38	34	52.8	5.3	2.43	0.8–7.35	0.12	1.77	0.52–6.03	0.36
Norovirus	78	40	38	51.3	4.9	1.68	0.64–4.41	0.29	1.29	0.44–3.74	0.64
*Shigella*	68	37	31	54.4	5.6	0.97	0.29–3.27	0.96	0.98	0.28–3.34	0.97
No pathogen detected	145	75	70	51.7	4.0	1.09	0.47–2.54	0.85	1.11	0.47–2.6	0.82
ETEC	177	90	87	50.8	2.7	1.0 (ref)			1.0 (ref)		
Consumed cheese in the past week											
*Campylobacter*	62	50	12	80.6	3.1	0.39	0.11–1.41	0.15	0.39	0.1–1.46	0.16
*Cryptosporidium*	74	61	13	82.4	2.6	1.31	0.14–11.9	0.81	1.24	0.13–12.3	0.85
EAEC	34	28	6	82.4	0.0	0.67	0.1–4.46	0.68	0.66	0.09–4.98	0.69
*Giardia*	76	62	14	81.6	2.6	0.32	0.07–1.55	0.16	0.45	0.07–2.67	0.38
Norovirus GI	69	57	12	82.6	1.4	1.06	0.11–9.97	0.96	0.94	0.1–9.16	0.96
Norovirus GII	75	59	16	78.7	1.3	0.52	0.16–1.68	0.27	0.65	0.18–2.38	0.52
Norovirus	81	64	17	79.0	1.2	0.60	0.2–1.82	0.37	0.71	0.22–2.32	0.57
*Shigella*	70	58	12	82.9	2.8	***			***		
No pathogen detected	147	115	32	78.2	2.6	0.60	0.19–1.9	0.39	0.60	0.19–1.91	0.39
ETEC	179	137	42	76.5	1.6	1.0 (ref)			1.0 (ref)		
Consumed tap water in the past week											
*Campylobacter*	61	8	53	13.1	4.7	2.32	0.51–10.4	0.28	2.22	0.49–10.2	0.30
*Cryptosporidium*	73	9	64	12.3	3.9	1.21	0.13–11.4	0.87	0.64	0.16–17.1	0.68
EAEC	33	2	31	6.1	2.9	***			***		
*Giardia*	75	8	67	10.7	3.8	***			***		
Norovirus GI	68	6	62	8.8	2.9	***			***		
Norovirus GII	73	9	64	12.3	3.9	2.61	0.62–11	0.19	3.00	0.58–15.5	0.19
Norovirus	79	9	70	11.4	3.7	1.87	0.46–7.65	0.39	1.97	0.42–9.18	0.39
*Shigella*	69	8	61	11.6	4.2	0.58	0.07–5.2	0.63	0.59	0.07–5.26	0.63
No pathogen detected	142	20	122	14.1	6.0	1.25	0.34–4.63	0.74	1.27	0.34–4.75	0.72
ETEC	173	23	150	13.3	4.9	1.0 (ref)			1.0 (ref)		
Consumed milk in the past week											
*Campylobacter*	63	26	37	41.3	1.6	1.25	0.43–3.66	0.68	1.26	0.42–3.74	0.68
*Cryptosporidium*	75	31	44	41.3	1.3	0.21	0.02–1.85	0.16	0.17	0.02–1.58	0.12
EAEC	34	11	23	32.4	0.0	0.51	0.09–2.98	0.45	0.44	0.07–2.71	0.37
*Giardia*	77	33	44	42.9	1.3	1.38	0.32–5.98	0.67	1.38	0.28–6.69	0.69
Norovirus GI	69	28	41	40.6	1.4	1.52	0.28–8.14	0.63	1.42	0.26–7.84	0.68
Norovirus GII	74	32	42	43.2	2.6	1.25	0.44–3.57	0.67	1.38	0.42–4.49	0.59
Norovirus	80	35	45	43.8	2.4	1.28	0.5–3.32	0.61	1.42	0.5–4.01	0.51
*Shigella*	71	30	41	42.3	1.4	1.21	0.36–4.07	0.75	1.20	0.36–4.06	0.77
No pathogen detected	148	72	76	48.6	2.0	2.02	0.83–4.88	0.12	2.11	0.87–5.14	0.10
ETEC	178	83	95	46.6	2.2	1.0 (ref)			1.0 (ref)		
Consumed ice with drinks in the past week											
*Campylobacter*	61	23	38	37.7	4.7	0.95	0.31–2.91	0.93	1.05	0.33–3.29	0.94
*Cryptosporidium*	73	29	44	39.7	3.9	2.19	0.45–10.6	0.33	1.98	0.39–9.95	0.41
EAEC	34	14	20	41.2	0.0	1.20	0.26–5.59	0.82	0.94	0.18–4.93	0.94
*Giardia*	75	28	47	37.3	3.8	0.21	0.03–1.82	0.16	0.13	0.01–1.32	0.08
Norovirus GI	68	25	43	36.8	2.9	0.32	0.04–2.88	0.31	0.30	0.03–2.75	0.29
Norovirus GII	74	27	47	36.5	2.6	0.92	0.31–2.67	0.87	0.88	0.28–2.8	0.83
Norovirus	80	28	52	35.0	2.4	0.76	0.28–2.05	0.58	0.73	0.25–2.08	0.55
*Shigella*	69	27	42	39.1	4.2	0.64	0.18–2.32	0.50	0.63	0.17–2.32	0.49
No pathogen detected	148	63	85	42.6	2.0	1.01	0.43–2.39	0.98	1.00	0.42–2.42	0.99
ETEC	180	71	109	39.4	1.1	1.0 (ref)			1.0 (ref)		
Consumed ice cream in the past week											
*Campylobacter*	61	26	35	42.6	4.7	1.11	0.37–3.37	0.85	1.23	0.39–3.88	0.72
*Cryptosporidium*	73	30	43	41.1	3.9	1.08	0.22–5.24	0.92	0.95	0.19–4.82	0.95
EAEC	34	14	20	41.2	0.0	0.31	0.05–1.79	0.19	0.33	0.05–2.14	0.25
*Giardia*	75	28	47	37.3	3.8	0.21	0.03–1.82	0.16	0.15	0.02–1.51	0.11
Norovirus GI	67	23	44	34.3	4.3	0.95	0.16–5.63	0.96	0.93	0.15–5.57	0.94
Norovirus GII	73	25	48	34.2	3.9	0.77	0.25–2.33	0.64	0.58	0.16–2.03	0.39
Norovirus	79	27	52	34.2	3.7	0.80	0.29–2.17	0.66	0.71	0.24–2.08	0.53
*Shigella*	69	28	41	40.6	4.2	0.90	0.26–3.09	0.86	0.90	0.26–3.1	0.86
No pathogen detected	149	48	101	32.2	1.3	0.40	0.17–0.94	0.04	0.40	0.17–0.96	0.04
ETEC	180	57	123	31.7	1.1	1.0 (ref)			1.0 (ref)	–	
Consumed food from a reheated buffet in the past week											
*Campylobacter*	61	21	40	34.4	4.7	1.62	0.53–4.98	0.40	1.80	0.57–5.69	0.32
*Cryptosporidium*	73	27	46	37.0	3.9	5.00	0.9–27.9	0.07	4.91	0.86–27.9	0.07
EAEC	34	10	24	29.4	0.0	2.53	0.51–12.6	0.26	3.93	0.63–24.7	0.14
*Giardia*	75	29	46	38.7	3.8	2.99	0.66–13.6	0.16	3.07	0.61–15.5	0.17
Norovirus GI	68	24	44	35.3	2.9	1.95	0.36–10.5	0.44	1.96	0.36–10.8	0.44
Norovirus GII	72	21	51	29.2	5.3	0.10	0.01–0.91	0.03	0.07	0.01–0.71	0.02
Norovirus	78	24	54	30.8	4.9	0.34	0.1–1.14	0.08	0.36	0.1–1.29	0.12
*Shigella*	69	25	44	36.2	4.2	1.67	0.49–5.67	0.41	1.67	0.49–5.71	0.41
No pathogen detected	141	55	86	39.0	6.6	2.48	0.93–6.62	0.07	2.47	0.92–6.64	0.07
ETEC	171	64	107	37.4	6.0	1.0 (ref)			1.0 (ref)		
Consumed chicha in the past week											
*Campylobacter*	61	11	50	18.0	4.7	1.33	0.34–5.24	0.68	1.43	0.35–5.77	0.62
*Cryptosporidium*	73	17	56	23.3	3.9	5.44	1.08–27.3	0.04	5.78	1.08–30.9	0.04
EAEC	33	7	26	21.2	2.9	7.33	1.17–46.1	0.03	9.26	1.08–79.2	0.04
*Giardia*	75	14	61	18.7	3.8	***			***		
Norovirus GI	67	13	54	19.4	4.3	1.04	0.11–10.2	0.97	1.08	0.11–10.9	0.95
Norovirus GII	74	15	59	20.3	2.6	0.98	0.27–3.52	0.97	0.98	0.26–3.71	0.97
Norovirus	79	16	63	20.3	3.7	0.98	0.3–3.19	0.97	0.89	0.26–3.04	0.85
*Shigella*	69	14	55	20.3	4.2	1.23	0.29–5.23	0.78	1.23	0.29–5.25	0.78
No pathogen detected	142	34	108	23.9	6.0	1.92	0.61–6.02	0.26	1.91	0.61–6.02	0.27
ETEC	172	42	130	24.4	5.5	1.0 (ref)			1.0 (ref)		
Consumed street vendor food in the past week											
*Campylobacter*	63	17	46	27.0	1.6	0.86	0.26–2.89	0.81	0.89	0.26–3.08	0.86
*Cryptosporidium*	75	21	54	28.0	1.3	0.40	0.05–3.54	0.41	0.37	0.04–3.33	0.37
EAEC	34	8	26	23.5	0.0	0.32	0.03–3.06	0.32	0.27	0.02–2.96	0.28
*Giardia*	77	20	57	26.0	1.3	***			***		
Norovirus GI	70	17	53	24.3	0.0	***			***		
Norovirus GII	76	16	60	21.1	0.0	0.14	0.02–1.17	0.07	0.14	0.02–1.21	0.07
Norovirus	82	16	66	19.5	0.0	0.11	0.01–0.88	0.04	0.11	0.01–0.87	0.04
*Shigella*	71	20	51	28.2	1.4	0.64	0.16–2.6	0.53	0.67	0.16–2.78	0.58
No pathogen detected	146	52	94	35.6	3.3	1.30	0.52–3.24	0.57	1.36	0.54–3.4	0.52
ETEC	178	49	129	27.5	2.2	1.0 (ref)			1.0 (ref)		
Consumed unpeeled fruit in the past week											
*Campylobacter*	61	12	49	19.7	4.7	1.13	0.3–4.35	0.86	1.24	0.31–4.92	0.76
*Cryptosporidium*	73	17	56	23.3	3.9	0.52	0.06–4.66	0.56	0.47	0.05–4.28	0.50
EAEC	34	6	28	17.6	0.0	3.67	0.58–23	0.17	2.85	0.43–18.9	0.28
*Giardia*	75	18	57	24.0	3.8	1.06	0.2–5.79	0.94	0.75	0.12–4.68	0.76
Norovirus GI	68	15	53	22.1	2.9	1.89	0.31–11.4	0.49	1.68	0.27–10.5	0.58
Norovirus GII	73	20	53	27.4	3.9	1.84	0.6–5.65	0.29	1.89	0.55–6.43	0.31
Norovirus	79	22	57	27.8	3.7	1.77	0.63–4.96	0.27	1.82	0.61–5.43	0.28
*Shigella*	69	16	53	23.2	4.2	1.63	0.43–6.22	0.48	1.67	0.42–6.68	0.47
No pathogen detected	146	41	105	28.1	3.3	2.45	0.79–7.62	0.12	2.44	0.78–7.63	0.12
ETEC	177	50	127	28.2	2.7	1.0 (ref)			1.0 (ref)		

CI = confidence interval; EAEC = enteroaggregative *Escherichia coli*; ETEC = enterotoxigenic *E. coli*; OR = odds ratio.

* List-wise deletion was used to handle missing variables, and the asterisk (***) indicates that exposure to a risk factor was a perfect predictor of the outcome.

† Adjusted for age and gender.

‡ The number of cases of both ETEC and the respective pathogen (or cases without pathogen identified, as relevant) included in each case–case variable.

§ “Unknown” calculated with denominator of number of observations for each outcome variable.
